# The Management of Cases of Chronic Aural Suppuration

**Published:** 1909-01-30

**Authors:** 


### Otology.

### THE MANAGEMENT OF CASES OF CHRONIC AURAL SUPPURATION.

While there is no doubt that if all cases of acute and subacute otitis received adequate treatment in the initial stage they would rarely become chronic, it is, nevertheless, unfortunately true that chronic aural discharge is enormously prevalent among all classes of the population, and that there is great difficulty in properly dealing with this important and dangerous affection. Otologists are fairly well agreed on the general principles of treatment, and the main difficulties appear when the attempt is made to enforce these principles in individual cases, and especially when a conscientious effort is made to extend thorough treatment to the very large class of patients among the poorer of the population.

Treatment consists simply in disinfecting the aural cavities and in keeping them clean. The providing of adequate drainage is necessarily included in this programme; if caries of the bony walls or ossicles has occurred, it is further necessary to scrape away or otherwise thoroughly remove the diseased parts. In some cases curettage through the meatus, with or without removal of the ossicles, is sufficient to procure this result, and is followed by healing. If, however, the bony walls of the antrum are diseased, or if the mastoid cells are affected to such an extent that free drainage from them is impossible, then it is necessary to obtain access to the diseased parts and to perform the radical mastoid operation. In such cases the tympanic cavity is nearly always much diseased, and, as the upper part of this cavity is subdivided into numerous spaces by reflections of the mucous membrane around the ossicles and their ligaments, free drainage is not readily provided; it is therefore not generally thought advisable to leave the ossicles and membrana tympani in cases where opening of the antrum is indicated. Even were the tympanic disease to subside, the resulting adhesions are not likely to favour good hearing power. There are, then, three main forms of treatment of varying severity; simple disinfection, intrameatal removal of disease, and the radical mastoid operation. Many cases can be cured by the first method, properly and persistently carried out, a further number heal after the second form of procedure, while others cannot get well until the radical mastoid operation has been performed.

*Simple Disinfection.*—In most cases with profuse discharge a syringe should be used. The effect of syringing is chiefly the mechanical removal of discharge, and it is generally advisable to use only mild antiseptics in this way. The syringing must be done properly and in the direction of the meatus; the nozzle must not fit the canal tightly, and no force should be used. The lotion should always be warm, its temperature should be 100° F., and the ear must be carefully dried afterwards with pledgets of clean wool. The meatus should also be very lightly plugged with clean wool which is changed as soon as it becomes moist. It matters little what antiseptic be used as long as it is not too irritating: boracic lotion and 1 in 80 carbolic are useful, and it is often advantageous to change the lotion from time to time. The important points are to keep the ear as dry as possible, and to observe the strictest cleanliness to obviate reinfection. Drops are often of value, and may be used after syringing, or instead if the discharge is slight. Rectified spirit containing 10 grains of boric acid to the ounce is very useful, especially if granulations are present; carbolic acid, 20 grains to the ounce of glycerine may be tried, or zinc sulphate 4 grains to an ounce of spirit. Peroxide of hydrogen is valuable when the discharge is thick or foul. All these drops should be used warm. Spirit drops dry quickly, but the others should be mopped out after five minutes. The great point is to keep the ear dry and free from stagnant discharge, and in many cases the best treatment is simply frequent mopping with cotton-wool on a probe, but the wool must be properly introduced into the tympanum. Cases with scanty discharge often do well under the insufflation of fine dusting powders. Boric acid is the most often used, but is sometimes irritating. Many apparently intractable cases can be cured by these simple methods if only the principles of preventing reinfection and accumulation of discharges, that is of dryness and cleanliness, are thoroughly and perseveringly carried out; but this is where the practical difficulties appear. It is often necessary to repeat the treatment three or four times a day; strict surgical cleanliness is required, and treatment must be continued until healing is complete. It is frequently most difficult to obtain these requisite conditions among the better class of patients, and generally quite impossible with the poorer classes.

Among hospital patients the essential cleanliness is unattainable, and among the educated classes also it is often advisable for the case to be dressed daily or at frequent intervals by the medical attendant; here the question of expense stands in the way, for the treatment may have to be continued for an indefinite, often a prolonged period, and may finally prove unsuccessful, so that operative measures are after all needed.

There are no means of providing skilled attention once a day in our hospitals for all cases of chronic aural discharge. In view of the recently instituted medical inspection of schools this problem is assuming a larger importance, and perhaps the establishment of school clinics will be able to deal with this class of case in children of the school age, though it will in no way help in the treatment of younger and older patients. In private practice, too, it is most difficult to persuade patients to give the necessary time and attention. The public are very ignorant of the dangers of discharge from the ears, and disinclined to give the trouble necessary for effective treatment. It is the plain duty of the medical profession to educate public opinion in this matter. The fact that some of the leading insurance companies refuse to accept the lives of those suffering from aural discharge on the usual terms should have some weight.

